# Cross-Amplification and Validation of SNPs Conserved over 44 Million Years between Seals and Dogs

**DOI:** 10.1371/journal.pone.0068365

**Published:** 2013-07-16

**Authors:** Joseph I. Hoffman, Michael A. S. Thorne, Rob McEwing, Jaume Forcada, Rob Ogden

**Affiliations:** 1 Department of Animal Behaviour, University of Bielefeld, Bielefeld, North Rhine-Westphalia, Germany; 2 British Antarctic Survey, Natural Environment Research Council, High Cross, Cambridge, United Kingdom; 3 Wildgenes Laboratory, Royal Zoological Society of Scotland, Edinburgh, United Kingdom; University of Sydney, Australia

## Abstract

High-density SNP arrays developed for humans and their companion species provide a rapid and convenient tool for generating SNP data in closely-related non-model organisms, but have not yet been widely applied to phylogenetically divergent taxa. Consequently, we used the CanineHD BeadChip to genotype 24 Antarctic fur seal (*Arctocephalus gazella*) individuals. Despite seals and dogs having diverged around 44 million years ago, 33,324 out of 173,662 loci (19.2%) could be genotyped, of which 173 were polymorphic and clearly interpretable. Two SNPs were validated using KASP genotyping assays, with the resulting genotypes being 100% concordant with those obtained from the high-density array. Two loci were also confirmed through *in silico* visualisation after mapping them to the fur seal transcriptome. Polymorphic SNPs were distributed broadly throughout the dog genome and did not differ significantly in proximity to genes from either monomorphic SNPs or those that failed to cross-amplify in seals. However, the nearest genes to polymorphic SNPs were significantly enriched for functional annotations relating to energy metabolism, suggesting a possible bias towards conserved regions of the genome.

## Introduction

Single nucleotide Polymorphisms (SNPs) are increasingly popular tools for population genetic studies of natural populations, but can be difficult to develop in non-model organisms due to a paucity of genomic information [1]. However, recent studies have shown that high-density SNP arrays developed for humans and domestic species including the chicken, cow, horse, pig, sheep and dog [2] can be successfully co-opted for use in closely related non-model organisms, in which they can yield large numbers of markers for a relatively modest technical effort and expenditure [3]–[7]. Few studies have so far extended this approach to species that are phylogenetically more distant to those in which the arrays were originally developed, probably because the proportion of SNPs remaining polymorphic is expected to decline rapidly with phylogenetic distance, dropping to around 5% for species that have diverged three million years ago [7]. Nevertheless, given a large enough number of loci on the initial array, even a tiny proportion of cross-amplifying SNPs may amount to a useful panel of markers for species that completely lack genomic resources. This assertion is supported by a recent study that cross-amplified SNPs from the BovineSNP50 BeadChip in Oryx species, which are divergent from *Bos* by around 23 million years, to obtain 149 polymorphic loci [8].

As with other markers such as microsatellites, a common problem with SNP discovery is that it can be prone to ascertainment bias. For example, both the size of the discovery panel of individuals and whether or not a SNP originates from a coding or non-coding region can influence minor allele frequencies, leading to downstream biases in population genetic estimates such as *F*_st_ [9]. One possibility that has been acknowledged but little evaluated is that SNPs cross-amplifying from high-density arrays could be enriched for conserved genomic regions that retain ancestral polymorphisms [5], some of which could potentially be subject to balancing selection [10]. Set against this, however, it is believed that the majority of SNPs on commercially available arrays are selectively neutral, since the loci are typically selected to provide even genomic coverage [7].

SNPs are increasingly being developed for use in marine mammals, where they have already provided insights into the population structure of bowhead and sperm whales [11], [12]. Because most SNP genotyping platforms only require around 120 bp of flanking sequence, SNPs are also ideally suited to genotyping historical or degraded samples such as whale bone or baleen [13], thereby facilitating new avenues of research. However, SNP development in marine mammals has so far largely proceeded along traditional lines, i.e. Sanger sequencing fragments derived from random genomic libraries or PCR amplified using conserved mammalian primers [11], [14]. These approaches are reliable but labour intensive, constraining the number of loci that can be developed (e.g. 18 in Sperm whales [15] and 42 in Bowhead whales [11], [15]).

An alternative approach to SNP discovery, facilitated by emerging high-throughput sequencing technologies, is to develop a transcriptome, which can be interrogated bioinformatically to identify thousands of genetic markers. This was recently done for the Antarctic fur seal (*Arctocephalus gazella*), a sexually dimorphic pinniped that has been intensively studied for several decades at Bird Island, South Georgia. To improve genetic resolution for ongoing studies of reproductive success [16], mate choice [17] and heterozygosity-fitness correlations [18]–[21], we constructed a transcriptome assembly from non-destructively obtained skin biopsy samples [22]. Homology to the dog (*Canis lupus familiaris*) genome was then exploited to map transcripts to specific chromosomes, allowing development of a genome-wide distributed panel of 104 polymorphic SNPs [23]. We have since expanded the original transcriptome to incorporate different types of tissue obtained at necropsy from animals that died of natural causes [24], allowing more than 9,300 SNPs to be identified. However, it would be desirable to develop additional SNPs, ideally also from non-coding regions of the genome.

The aim of this study was to explore the cross-amplification utility of the CanineHD BeadChip, which carries a total of 172,662 canine SNPs, in the Antarctic fur seal. A total of twenty four fur seal individuals were therefore screened in order to ascertain which SNPs could be successfully genotyped and to identify polymorphic loci. We also explored the potential for bias in SNPs conserved between seals and dogs with respect to their genomic distribution, proximity to known genes and the functional annotations of nearby genes.

## Materials and Methods

### Tissue Sampling and DNA Extraction

Skin biopsy samples were collected from 24 unrelated Antarctic fur seal individuals (9 adult males, 13 adult females and 2 pups) during the austral summers of 2009/2010 and 2010/2011 at Bird Island, South Georgia (54° 00′ S, 38° 02′ W) using protocols described in detail by Hoffman *et al*. [16]. Skin samples were transferred to Dimethyl Sulphoxide (DMSO) saturated with salt and stored individually at −20°C. Total genomic DNA was extracted using an adapted Chelex 100 protocol [25] followed by phenol-chloroform purification [26]. Each sample was then quantified using a NanoView spectrophotometer (Fisher Scientific). DNA concentrations averaged 323 ng/µl and ranged from 155 to 778 ng/µl.

### Ethical Note

Tissue samples were collected by one of the authors (JF) as part of the Long Term Monitoring and Survey project of the British Antarctic Survey that has employed consistent sampling protocols since 1994. Tissues were obtained from adult males using standard protocols for remote biopsy sampling that have no known deleterious effects on the study animals. Sampling was authorised by the Senior Executive and the Environment Officers of the Government of South Georgia and the South Sandwich Islands, and samples were collected under Scientific Research Permits for the British Antarctic Survey field activities on South Georgia during the 2009/10, 2010/11 seasons. Tissue samples were collected and retained under permits issued by the Department for Environment, Food and Rural Affairs (license number AHZ/2024A/2005/1) and in accordance with the Convention on International Trade in Endangered Species of Wild Fauna and Flora (permit numbers 004/2011 and 464895/04). All procedures used were approved the British Antarctic Survey Ethics Committee (reference number PEA6), which includes members of Cambridge University.

### CanineHD BeadChip Genotyping

The samples were genotyped using the Illumina canine high density SNP chip which enables the simultaneous genotyping of 172,662 SNP markers identified from CanFam2.0, the second build of the dog genome reference sequence [27]. The SNPs were selected to represent as many different dog breeds as possible while providing even coverage of the genome. Validation across 26 breeds identified a total of 143,889 polymorphic SNPs (range = 85,193–126,387) with an average call rate of 99.8% [28]. The seal samples were genotyped following recommended assay protocols with bead chips scanned using the Illumina iScan platform.

### Scoring the SNP Data

Automated allele calling was implemented using the software GenomeStudio 2010.1 (Genotyping module 1.7.4 version 2011.1, Illumina). This program normalizes the intensity data for each of the loci and then assigns each sample a cluster position. The resulting genotype output is then provided together with two quality measures, the GenTrain and GenCall scores [29]. The GenTrain score is a locus-specific measure that takes into account the quality and shape of the genotype clusters and their relative distances from one another. The GenCall score, estimated for each individual at each SNP, provides a measure of the proximity of each genotype to the centre of clusters, with those located further away being considered less reliable. We only accepted loci with a GenTrain score ≥0.25 and only called individual genotypes with GenCall scores ≥0.25. These represent stringent thresholds previously applied in studies of humans [29] and other species [30], [31], [32]. We also checked all of the scores manually within GenomeStudio and made minor adjustments to the clustering where necessary following Hoffman et al. [23].

### Data Analysis

Identification of SNP markers in the seal samples was made on the basis of different genotype clusters observed within the Genome Studio software. Where two alleles were clearly observed, either in a heterozygous or homozygous state, the marker was categorized as a polymorphic SNP in seals. Where fluorescence intensity readings indicated the presence of a single allele across all samples (normalised R >0.1), the marker was designated as monomorphic. This is based on the assumption that the observed data reflect amplification of a homologous sequence region in the seal genome that did not exhibit polymorphism at the nucleotide targeted by the canine assay. Genepop [33] was then used to calculate observed and expected heterozygosities and to test for deviations from Hardy-Weinberg equilibrium (HWE) and for linkage disequilibrium (LD) among markers. The resulting *P*-values were adjusted for the false discovery rate [34] using the program Q-value [35].

### Bioinformatic Analyses

The full set of SNPs (those that failed to amplify, the monomorphic and the polymorphic with respect to *A. gazella*) with 120 bp flanking sequence were mapped to the dog genome (Broad Institute release 67) and the *Arctocephalus* transcriptome [24] using Blast [36]. When mapping against the genome, the most proximal gene to each SNP was selected and, through Swissprot [37], the Gene Ontology (GO) codes [38] for each gene were derived. Each GO code was tested for enrichment through a ratio test in the polymorphic set against the monomorphic SNPs as well as the SNPs that failed to hybridize with *A. gazella*. A subsequent adjustment of the *P*-values was applied following Benjamini and Hochberg [34].

### *In vitro* SNP Validation

In order to validate the cross-amplification SNP discovery process, a subset of five randomly selected loci were targeted for confirmatory genotyping using single-plex KASP assays (LGC Genomics), which are based on fluorescently labeled allele-specific PCR primers. Assays were designed based on flanking regions in the dog genome (Table S1). Concordance between the two genotyping methods would provide validation of the SNP. However, failure of the KASP assay need not necessarily refute the presence of a SNP, since KASP and Illumina assays may target different SNP flanking regions. The 24 fur seal samples together with six positive control (dog) samples and two negative controls (water) were genotyped following standard KASP PCR protocols.

## Results

**Figure 1:**
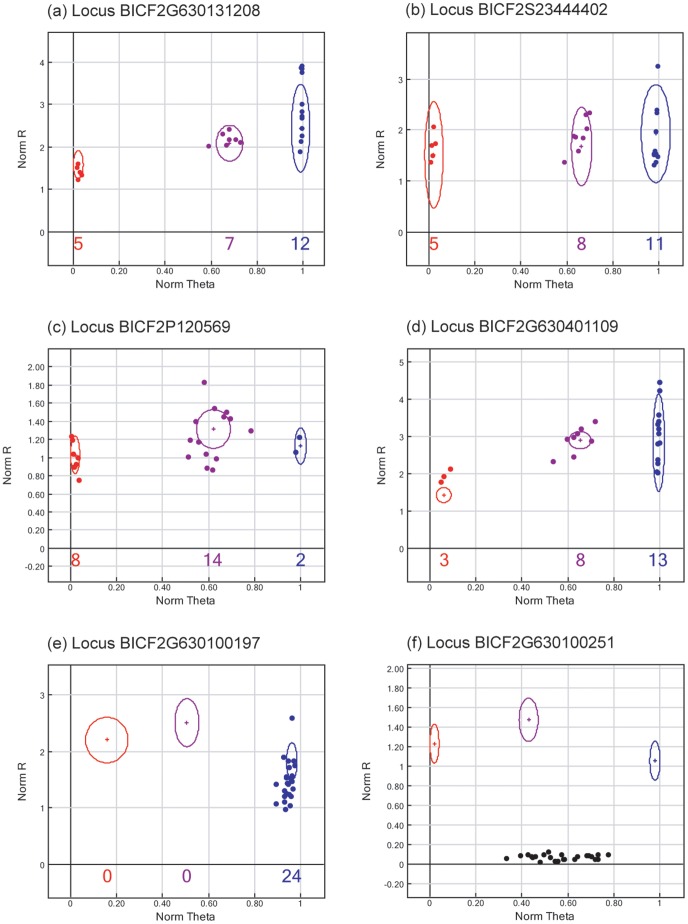
Examples of single nucleotide polymorphisms (SNPs) from the canine SNP chip that cross-amplify in Antarctic fur seals. Each point represents a single sample. ‘Norm R’ (y-axis) is the normalized sum of the intensities of the two channels (Cy3 and Cy5). ‘Norm Theta’ (x-axis) is ((2 / p)Tan)1 (Cy5 / Cy3)) where a value near 0 represents a homozygote for allele A (denoted by red points) and a value near 1 represents a homozygote for allele B (denoted by blue points). Heterozygotes fall approximately mid-way between these values and are denoted by purple points. The numbers of samples called by GenomeStudio for each of the three possible genotypes are shown below the x-axis. (a–d) Classical three-cluster patterns for SNPs considered successful and polymorphic; (e) A monomorphic SNP; (f) A locus that failed to yield an interpretable assay and was thus classified as a genotyping failure.

Out of a total of 173,662 loci on the CanineHD BeadChip, 33,324 (19.2%) were genotyped in a sample of 24 Antarctic fur seals (Table S2). Of these, 173 (0.5%) exhibited clearly interpretable polymorphic clustering patterns (see Fig. 1, panels a–d for examples) and correspondingly high GenTrain scores (mean = 0.77±0.07 s.d.). An additional twenty loci were polymorphic but could not be reliably scored due to ambiguous clustering patterns.

### Descriptive Statistics

Raw genotypes of the 173 clearly interpretable polymorphic SNPs in 24 Antarctic fur seal individuals are given in Table S3. Twelve of these loci (6.9%) deviated significantly from HWE at *P*<0.05, although only six remained significant following table-wide correction for the false discovery rate (see Table S4). The call rate ranged from 0.875 to 1 (mean = 0.99±0.02 s.d.) and the minor allele frequency varied between 0.02 and 0.50 (mean = 0.17±0.13 s.d.). Tests for linkage disequilibrium (LD) did not yield any *P*-values that were robust to table-wide correction for the false discovery rate.

### Mapping Loci to the *Arctocephalus* Transcriptome

We first mapped all of the SNPs to the *Arctocephalus gazella* transcriptome, which comprises 23,096 contigs of average length 971 bp with a combined length of 22,425,629 bp [24]. BLAST hits to seal transcripts were recovered for 3.6% of polymorphic SNPs (*n* = 7) and 4.1% of monomorphic SNPs (*n* = 1367) but only 1.0% of failed SNPs (*n* = 1467), significantly lower than for the previous two classes (Binomial proportions tests, *P* = 0.002 and *P*<0.0001 respectively). When this analysis was repeated with the stringent requirement of at least 110 bp of sequence overlap, fewer than half as many mappings were obtained but the overall pattern was similar, BLAST hits being obtained for two polymorphic SNPs (1.0%), 659 monomorphic SNPs (2.0%) and 580 failed SNPs (0.41%).

We next attempted to verify *in silico* the seven polymorphic SNPs revealing homology to the *Arctocephalus* transcriptome through visual inspection within the program Tablet [39]. In two cases, part of the flanking sequence mapped to a fur seal transcript but the SNP itself was intronic in the dog and so could not be found (Table 1). In a further four cases, no variation was present within the transcript at the location corresponding to the SNP, but the depth of coverage was almost certainly too low (mean = 3.75 reads, range = 1–6 reads) to be able to detect the polymorphism given the minor allele frequency observed in the fur seal. In the remaining two cases, where depth of coverage was much higher at 12x and 42x respectively, both SNPs were confirmed as being present within the *Arctocephalus* transcripts. Moreover, locus BICF2G630131208 had previously been independently called as a ‘high-quality SNP’ [24] by the Newbler mapping program, which requires at least three non-duplicate reads showing the variant and at least seven reads with Phred quality scores of at least 20.

**Table 1:** Details of seven polymorphic SNPs revealing homology to the *Arctocephalus gazella* transcriptome.

Locus name	Type	Minor allele frequency(MAF)	Contig number	Bitscore	E-value	Identity	Position of SNP incontig	Total depth of coverage at SNP position	Number of reads differing from the consensus
BICF2P1458746	[T/C]	0.10	AgU008421_v1.1	194	5e^−50^	110/114 (96%)	297	12	1
BICF2S22920466	[T/C]	0.13	AgU000126_v1.1	186	1e^−47^	112/118 (94%)	249	6	0
BICF2G630131208	[T/C]	0.35	AgU001255_v1.1	112	4e^−25^	65/68 (95%)	1427	42	10
BICF2G630805987	[T/C]	0.08	AgU010822_v1.1	88	9e^−18^	73/80 (91%)	NA	NA	NA
BICF2P1240299	[A/G]	0.50	AgU001844_v1.1	132	2e^−31^	83/90 (92%)	1875	4	0
BICF2P388183	[T/G]	0.08	AgU010256_v1.1	70	2e^−12^	41/43 (95%)	NA	NA	NA
TIGRP2P316903_rs8524601	[T/C]	0.44	AgU004294_v1.1	76	3e^−14^	60/66 (90%)	11	1	0

Minor allele frequency is derived from the observed genotypes of 24 Antarctic fur seal individuals. NA refers to loci for which part of the flanking sequence but not the SNP itself mapped to a fur seal transcript. Note that locus BICF2G630131208 was independently called as a ‘high-quality SNP’ by the Newbler mapping program [24]. For a SNP to be called in this way, there must be at least three non-duplicate reads showing the variant, with these reads being represented in both the forward and reverse directions, and at least seven reads with Phred quality scores of at least 20.

### *In vitro* SNP Validation

**Figure 2:**
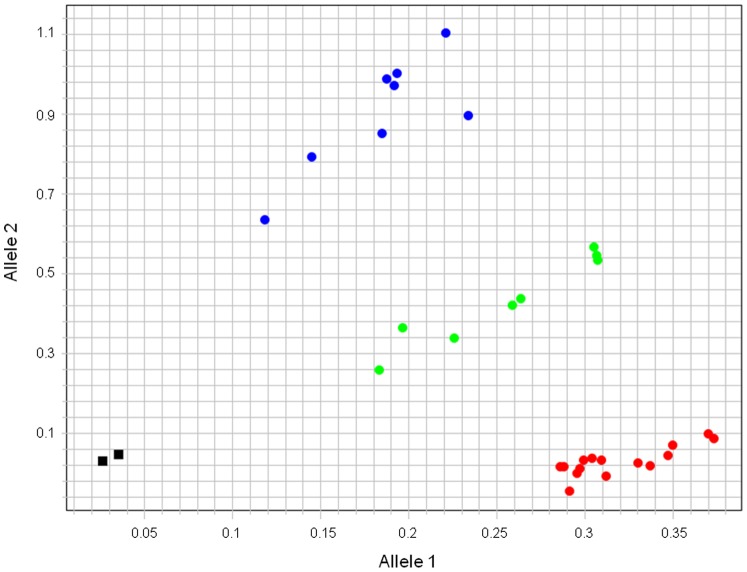
Example genotypes obtained for locus BICF2G630131208 using KASP chemistry and an ABI Step-One real-time PCR machine. The three discrete clusters of heterozygous and alternative homozygous genotypes denoted by green, red and blue points respectively include all 24 Antarctic fur seals as well as positive canine control samples. The two black squares indicate negative controls.

Two of the five KASP assays (BICF2G630131208 and BICF2G630510520) yielded identical genotypes to the Illumina array (Table S5), thereby validating the presence of these SNPs in the fur seal genome (see Figure 2 for an example). A third assay (BICF2G630401109) gave clear amplification results in 29% of the seal samples, although all of the genotypes at this locus were homozygous. The remaining two assays failed to amplify in fur seals. Positive dog controls and negative controls gave expected results for all SNPs.

### Mapping and Enrichment Analyses

**Figure 3:**
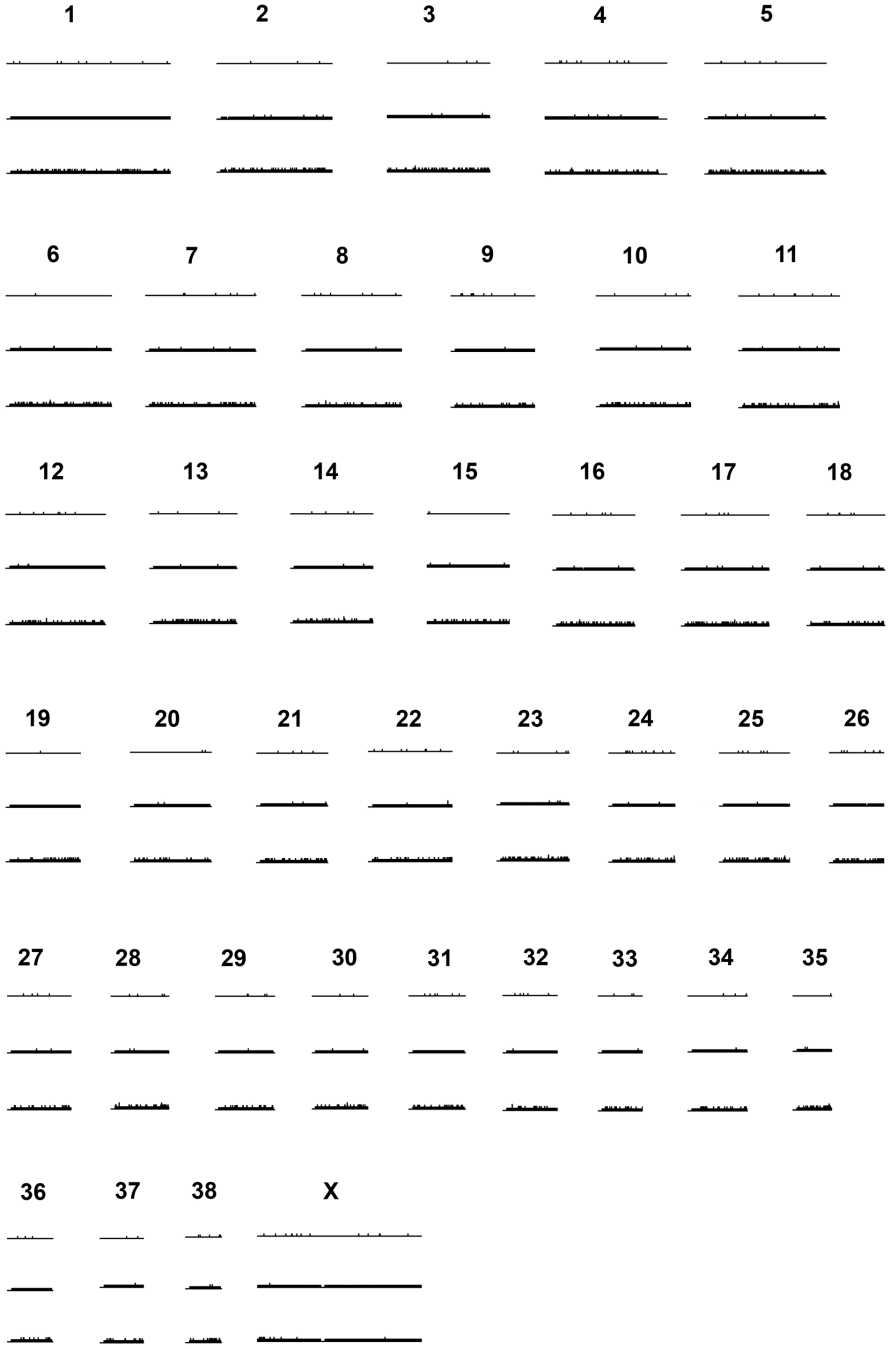
Distribution of polymorphic, monomorphic and failed SNPs mapped to the dog (***Canis lupus familiaris*** **) genome (shown as three rows,** ***n*** **= 193,** ***n*** **= 33,131 and** ***n*** **= 136,903 respectively).**

**Figure 4:**
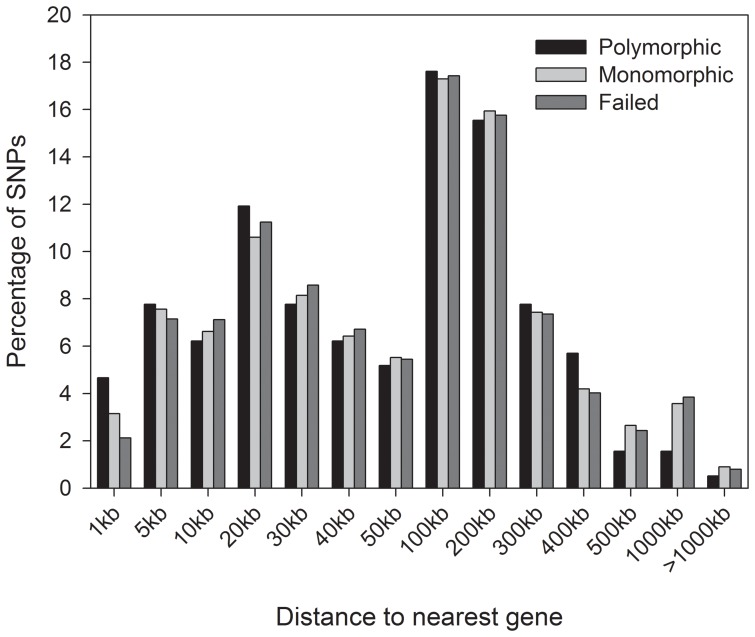
Distribution of the distance between each SNPs and its nearest gene in the dog, shown for polymorphic, monomorphic and failed SNPs.

We next explored the genomic distribution of SNPs in the dog (*Canis lupis familiaris*). A GFF file containing details of the working SNPs together with their locations relative to the dog genome is provided that allows these data to be viewed as an additional track within Ensembl (File S1). No obvious differences were observed between polymorphic SNPs, monomorphic SNPs and those that failed to cross-amplify, either in terms of chromosomal location or in relation to gene density (Figure 3). Moreover, distances between SNPs and their nearest genes did not vary significantly among the three classes of loci (Figure 4, Kruskal-Wallis rank sum test, χ^2^ = 0.85, df = 2, *P* = 0.65). We therefore conducted an enrichment analysis to test for any differences in the functional annotations of these genes, based on a total of 5581 GO categories represented in the full dataset. Seven GO terms were nominally identified as being enriched in the genes nearest to polymorphic SNPs in comparison to monomorphic SNPs, based on an adjusted *P*-value threshold of 0.05 (Table S6). However, these could be type I errors because the *P*-values were marginal, reflecting the presence/absence of a single gene. A further GO term entitled ‘generation of precursor metabolites and energy’ (GO:0006091) was significantly enriched in the genes nearest to polymorphic SNPs relative to both monomorphic and failed SNPs (adjusted *P*-values were both <0.0001), with this inference being based upon the presence/absence of 26 different genes.

## Discussion

Several recent studies have exploited high-density SNP arrays developed for model organisms to obtain genetic markers for closely related non-model species, but relatively few have applied this approach to more distantly related taxa. Here we show that, despite seals and dogs having diverged around 44 million years ago, genotype data could be obtained for 173 polymorphic loci, a small subset of which were independently validated using a combination of *in vitro* and *in silico* approaches.

### Cross-amplification Rate

Over 33,000 SNPs from the CanineHD array cross-amplified in the Antarctic fur seal, a number that although large represents only 19.2% of the total number of SNPs evaluated. This is around 10% lower than predicted by a linear regression of the percentage of SNPs amplified on the time to last common ancestor, based on data from 117 species genotyped on ovine, bovine and equine SNP50 bead chips [7]. One explanation for this shortfall could relate to the observation that, when the CanineHD array was evaluated in 26 dog breeds, only 82.9% of SNPs (*n* = 143, 889) were found to be polymorphic [28], perhaps suggestive of a moderate rate of technical failure. Another contributing factor could be that the CanineHD array was generated from numerous different dog breeds, meaning that many of the SNPs could be relatively recent. To explore this further would require knowledge of which of the SNPs are specific to particular breeds.

Despite the overall cross-amplification rate being somewhat lower than expected, we were nonetheless able to identify 193 polymorphic SNPs, all but twenty of which showed clearly interpretable clustering patterns. This is roughly an order of magnitude fewer SNPs than obtained for bison using the CattleSNP50 BeadChip [3], [5] and is 2−3 times less than obtained for bighorn and thinhorn sheep using the OvineSNP50 BeadChip [7]. However, this makes good sense because the percentage of amplified loci that remain polymorphic is known to decline exponentially with phylogenetic distance before leveling off after around five million years of divergence [7]. Consistent with this, a recent study that cross-amplified SNPs from the BovineSNP50 BeadChip in Oryx, which are divergent from *Bos* by around 23 million years, obtained a comparable 149 polymorphic loci [8]. That we obtained more markers despite a substantially greater divergence time between seals and dogs presumably reflects the larger number of SNPs on the canine array. If so, the utility of high-density arrays for studying non-model organisms may depend not only upon phylogenetic distance, but also on array size.

### SNP Validation

Several recent studies have used high-density arrays developed in model species to cross-amplify SNPs in their wild counterparts [3]–[8]. However, only a single study has so far validated the resulting SNPs in the focal species, in this particular case by Sanger sequencing a handful of the loci [8]. We therefore explored the use of both *in vitro* and *in silico* approaches for confirming or refuting the presence of SNPs identified in the Antarctic fur seal. In the first of these, the results observed for the two KASP assays that displayed 100% concordance with the high density array confirmed the presence of both SNPs in the fur seal genome. However, a third locus partially amplified and the two remaining KASP assays completely failed. Taken at face value, this would imply a conversion rate of somewhere between 40 and 60%, although we believe it would be premature to draw firm conclusions based on a sample size of only five loci tested. If anything, our study highlights the difficulty of *in vitro* SNP validation for target loci that are typically too small to reliably sequence and which may differ significantly in genotyping assay conversion success owing to associated differences in primer/probe target sites.

Negative control samples (water) were not run on the canine SNP chip and spurious genotypes are sometimes generated by the Illumina Infinium chemistry in the absence of target template DNA. However, if we were witnessing spurious amplification that was not from seal DNA but from other constituents of the sample, we would not expect to observe a small subset of the same SNPs consistently amplifying across all 24 seal samples. Moreover, negative controls were included in the KASP assays and behaved as expected (see Figure 2). This is strongly suggestive of the amplification of seal template DNA for these loci. In addition, testing for deviations from HWE can provide an important means of quality control [40] capable of identifying loci that are not genuine SNPs, such as those residing within duplicated regions of the genome [41] as well as flagging up genotyping problems such as pipetting error, cross-contamination of samples and non-specificity or instability of the genotyping assay [42]. We therefore tested each of the 173 polymorphic SNPs for deviation from HWE using empirical data from 24 unrelated fur seal individuals. The results were promising in that only a handful of loci deviated significantly from HWE (6.9% prior to correction for multiple statistical tests). Further work could be undertaken to confirm that the alleles are segregating in a Mendelian fashion [3], for example by genotyping known fur seal mother-offspring-father triads on the canine array, although loci with low MAFs would require relatively large sample sizes in order to identify triads in which inheritance could be formally verified.

A recently developed transcriptome [24] also allowed us to confirm that two polymorphic SNPs were present in the fur seal through *in silico* visualisation. One of these loci had also previously been identified as being a ‘high-quality SNP’ during an independent round of marker discovery, indicating that this locus fulfils several stringent selection criteria. Finding such a match not only helps to confirm that the SNP in question is common to both dogs and seals, but it also implies that our transcriptome assembly is of reasonable quality. Unfortunately, our sample size of cross-amplified polymorphic SNPs was too small to allow larger numbers of SNPs to be similarly located. Nevertheless, our analysis suggests that transcriptome assemblies could potentially be of some value generally for SNP validation.

### Potential Biases in SNP Discovery

Consistent with the canine array having been designed to provide even genome-wide coverage, we found no obvious spatial clustering within the canine genome of SNPs that were polymorphic in Antarctic fur seals. Moreover, in support of a recent study that cross-amplified rhesus macaque SNPs in seven old world monkey species [9], we found no significant differences in proximity to nearest genes among polymorphic, monomorphic or SNPs that failed to genotype in fur seals. Nevertheless, we went a step further by exploring whether the closest genes to these three classes of SNP showed any obvious patterns of functional enrichment. With the exception of a small number of marginally significant GO terms that appear consistent with type I errors, we found only a single term that was significantly over-represented in genes proximal to polymorphic SNPs. The term in question, ‘generation of precursor metabolites and energy’, includes genes involved in fundamental energetic pathways including the electron transport chain. As is the case for metabolic genes in general [40] and given the essential role that these specific genes play in energy metabolism, it is plausible that they exhibit high levels of evolutionary sequence conservation, potentially helping to explain the retention of local SNPs. However, this analysis should be treated with caution due to the small sample size of polymorphic loci.

Balancing selection is a powerful force that might also help to explain why certain polymorphisms are retained over long timescales while others are not [41]. However, long-term balancing selection is generally considered to be rare, studies of humans having only identified a few tens to hundreds of genes that show the expected signatures [42]–[44]. Moreover, SNPs on commercial arrays are generally thought to be selectively neutral since they are usually chosen to provide even chromosomal coverage [7]. Unfortunately, very little is known about balancing selection in either dogs or seals, other than the fact that this may be operating at the canine MHC [45] and MC1R [46]. Moreover, classical tests for balancing selection cannot be applied in the context of this study because they require data on intraspecific and/or interspecific sequence variation [47] that are not available. Nevertheless, the 173 polymorphic SNPs were not tightly clustered around a small number of genes as would be expected, for example, if proximity to the MHC was of key importance. Moreover, none of the polymorphic SNPs reside within 1 Mb of any known MHC genes [24] nor MC1R in the dog genome. Thus, although our data do not provide the means to test decisively for balancing selection, we do not find any clear evidence pointing towards balancing selection being responsible for the retention of the SNPs identified in this study.

### Alternative Approaches for SNP Identification and Genotyping

The recent development of high-throughput sequencing approaches such as Roche 454 [48] and Illumina HiSeq [49] has made it possible to gather unprecedented amounts of genetic information from non-model organisms [50]. This has led to the widespread uptake of approaches such as transcriptome sequencing [51] and Restriction Site Associated DNA (RAD) sequencing [52]. Transcriptomes are particularly powerful resources because they can easily be mined *in silico* for SNPs, which in turn can be genotyped on whatever scale is required (reviewed by [53]). RAD sequencing can similarly be used for SNP discovery, or it can be employed for primary data collection. These and related approaches are extremely powerful because they are capable of generating vast amounts of genomic data for virtually any organism. However, as with any technique, they also have a number of drawbacks. For example, to assemble large volumes of transcriptome data and call SNPs requires access to computing infrastructure and bioinformatic expertise, while RAD sequencing, at least in the set-up stages, may require considerable wet-lab optimisation. High-density SNP arrays could therefore provide a viable alternative for certain species, particularly for conservation genetic projects involving small numbers of individuals, primarily because of their rapidity and ease of use. This project, for example, took just five days from DNA extraction to SNP calling, with the latter being conveniently implemented within Illumina’s user-friendly GenomeStudio software.

### Conclusion

We used the CanineHD beadArray to genotype 173 polymorphic SNPs in 24 Antarctic fur seal individuals. Although our efforts to validate a subset of SNPs met with mixed success, we nevertheless obtained 100% genotype concordance for two of the loci using KASP assays and also confirmed that two loci were present in fur seals using *in silico* mapping. The enrichment of polymorphic loci for proximity to genes involved in energy metabolism could potentially help to explain why some SNPs appear to be retained over long evolutionary timescales.

## Supporting Information

File S1 **Arctocephalus_SNPs.gff.** A GFF file containing details of the polymorphic SNPs together with information on their locations in relation to the dog genome (CanFam3.1) that can be viewed as a track within Ensembl.
(GFF)

Table S1 **Details of single-plex KASP assays (LGC Genomics) used to validate five putative fur seal SNPs.**
(XLSX)

Table S2 **Details of all 173,662 canine SNPs, characterised as polymorphic, monomorphic or failed in a sample of 24 Antarctic fur seals.** Asterisks denote twenty loci that were polymorphic but which could not be scored reliably. Included are the flanking sequences of all SNPs together with their chromosomal coordinates in the dog (*Canis lupis familaris*) genome and the identity of any *Arctocephalus gazella* transcripts to which a given SNP mapped.
(XLSX)

Table S3 **Genotypes generated for 173 clearly interpretable polymorphic SNPs in 24 Antarctic fur seals (see Results for details).**
(XLSX)

Table S4 **Polymorphism characteristics of 173 clearly interpretable polymorphic SNPs in 24 Antarctic fur seals (see Results for details).** The GenTrain score takes into account the quality, shape and degree of separation of the genotype clusters, with higher values indicating improved clustering [29]. *P*-values for deviation from HWE are shown without correction for multiple statistical tests. Values significant at *P*<0.05 are highlighted in bold, while those remaining significant after controlling for the false discovery rate are underlined. Twenty loci that were polymorphic but which could not be reliably scored are not included.
(XLSX)

Table S5 **Genotypes generated for five SNP loci using KASP assays in six dog and 24 Antarctic fur seal individuals.**
(XLSX)

Table S6 **Results of the enrichment analysis based on Gene Ontology (GO) codes of the nearest genes to each SNP (See Materials and methods for details).** Only GO codes that were significantly enriched in comparisons involving polymorphic with monomorphic or polymorphic with failed SNPs are shown, following *P*-value adjustment for multiple tests.
(XLSX)
